# Ceftazidime–Avibactam Use in a Case Series of Difficult-to-Treat or Recurrent Infections in Pediatric Patients with Complex Chronic Conditions: Effectiveness and Absence of Resistance Development

**DOI:** 10.3390/antibiotics13070598

**Published:** 2024-06-27

**Authors:** Miguel García-Boyano, María Alós Díez, Lorena Fernández Tomé, Luis Escosa-García, Francisco Moreno Ramos, Cristina Schuffelmann-Gutiérrez, Emilio Cendejas Bueno, Cristina Calvo, Fernando Baquero-Artigao, Esteban Frauca Remacha

**Affiliations:** 1 Pediatric Infectious Disease Department, La Paz University Hospital, 28046 Madrid, Spain; miguelgarciaboyano@gmail.com (M.G.-B.); ccalvorey@gmail.com (C.C.); fbaqueroartigao@gmail.com (F.B.-A.); 2 La Paz Research Institute—IdiPAZ, 28046 Madrid, Spain; maria.alosdiez@gmail.com (M.A.D.); lftome@salud.madrid.org (L.F.T.); fmorenor@salud.madrid.org (F.M.R.); ecendejas77@gmail.com (E.C.B.); esteban.frauca@salud.madrid.org (E.F.R.); 3 ERN-TransplantChild, Pediatric Hepatology Service, La Paz University Hospital, 28046 Madrid, Spain; 4 Área de Enfermedades Infecciosas del Centro de Investigación Biomédica en Red del Instituto de Salud Carlos III (CIBERINFEC), Instituto de Salud Carlos III, 28029 Madrid, Spain; 5 Department of Pharmacy, La Paz University Hospital, 28046 Madrid, Spain; 6 Pediatric Intensive Care Unit, La Paz University Hospital, 28046 Madrid, Spain; cschuffelmann@yahoo.es; 7 Clinical Microbiology Department, La Paz University Hospital, 28046 Madrid, Spain

**Keywords:** ceftazidime–avibactam, carbapenem-resistant *Enterobacteriaceae*, beta-lactamase, OXA-48, *Klebsiella pneumoniae*, child, liver transplantation, intraabdominal infections, cholangitis

## Abstract

The prevalence of multidrug-resistant Gram-negative infections, particularly carbapenem-resistant strains, has become a significant global health concern. Ceftazidime–avibactam (CZA) has emerged as a promising treatment option. However, data on its efficacy and safety in children are scarce, necessitating further investigation. We conducted a descriptive case series at a tertiary hospital in Spain from February 2019 to January 2022. Pediatric patients (<16 years) treated with CZA for confirmed or suspected multidrug-resistant Gram-negative infections were included. The clinical and microbiological characteristics, treatment approaches, and outcomes were examined. Eighteen children received CZA treatment. All had complex chronic conditions, with the most frequent underlying main diseases being liver transplantation (*n* = 8) and biliary atresia (*n* = 4). The predominant type of infection for which they received CZA was intra-abdominal infection caused or suspected to be caused by OXA-48-producing *Klebsiella pneumoniae*. CZA was generally well tolerated. Within the first month of starting CZA therapy, two patients died, with one case directly linked to the infection’s fatal outcome. Some patients needed repeated courses of therapy due to recurrent infections, yet no resistance development was noted. In summary, the use of CZA showed effectiveness and safety, while the lack of resistance development highlights CZA’s potential as a primary treatment option against OXA-48-producing infections.

## 1. Introduction

In recent decades, there has been a noticeable and widespread increase in the prevalence of multidrug-resistant (MR) Gram-negative infections, impacting both adults and children alike [1,2]. Recognizing the gravity of this escalating issue, the World Health Organization (WHO) issued its first-ever list of antibiotic-resistant priority pathogens in the year 2017. This comprehensive list pinpointed carbapenem-resistant (CR) and extended-spectrum β-lactamase (ESBL)-producing *Enterobacterales*, CR *Pseudomonas aeruginosa*, and CR *Acinetobacter baumannii* as constituting the most critical group [3]. Similarly, the Centers for Disease Control and Prevention (CDC) echoed these concerns by designating these pathogens as urgent and serious antibiotic resistance threats within the United States as of 2019 [4]. Notably, CR Gram-negative infections have been associated with elevated mortality rates in pediatric patients, often attributed to the limited treatment options available, delays in the initiation of appropriate antibiotic therapy, and the presence of underlying comorbidities [5,6,7].

While novel β-lactam agents designed to target CR organisms have obtained approval from the Food and Drug Administration (FDA) for use in adults [8], the dearth of pediatric-specific data pertaining to dosing, efficacy, and safety necessitates the prescribing of these medications off-label, with healthcare providers frequently relying on extrapolated data derived from studies conducted in adult populations.

In March 2019, the Food and Drug Administration (FDA) granted official approval for the utilization of CZA, which is a novel inhibitor of β-lactam–β-lactamase, for the purpose of treating complicated intra-abdominal infections (cIAI) when administered alongside metronidazole in children aged 3 months and older. Furthermore, it was also approved for the treatment of complicated urinary tract infections, which encompass pyelonephritis, in pediatric patients aged 3 months and above [9]. In September 2020, the European Medicines Agency (EMA) also approved its usage in pediatric patients aged 3 months and above for the management of hospital-acquired pneumonia, including cases of ventilator-associated pneumonia. Additionally, it was approved for addressing infections caused by aerobic Gram-negative organisms with limited treatment options [10]. Despite the aforementioned approvals from regulatory authorities, instances of clinical success in the management of CR infections in pediatric patients treated with CZA continue to be predominantly documented through isolated case reports and small case series [11]. It is noteworthy that ceftazidime displays comprehensive coverage against a multitude of Gram-negative organisms; however, its effectiveness against *Acinetobacter* spp. and strains that produce carbapenemase and ESBLs is notably limited. On the other hand, avibactam augments its effectiveness by impeding the activity of Ambler class A and class C β-lactamases, in addition to targeting a select class D enzymes, such as ESBLs, KPC, OXA-48 carbapenemases, and AmpC enzymes [10].

The primary objective of this study was to describe the clinical and microbiological characteristics, therapeutic approaches, and resultant outcomes observed in pediatric patients receiving treatment with CZA for confirmed or suspected (by prior colonization) MR Gram-negative infections within the confines of our medical facility. Special attention was devoted to examining the emergence of resistance to this antibiotic.

## 2. Results

### 2.1. Demographic Data, Risk Factors, and Clinical Presentation

During the time frame from February 2019 to January 2022, a total of 18 pediatric patients (comprising 11 males and 7 females) were administered CZA treatment for a minimum duration of 2 days at our medical institution. The median age at the commencement of treatment was 21 months (interquartile range [IQR] 10–43, range 87 days to 15.4 years).

Table 1 presents an overview of the complex chronic conditions observed in these 18 children, along with the risk factors associated with CR infection or colonization. Additionally, it provides details regarding the specific types of infections for which they received initial therapy with CZA, as well as information regarding their fever history, duration of illness, and diagnosis of sepsis and septic shock.

### 2.2. Laboratory Findings, Hospitalization, and Severity of Illness

The highest levels of C-reactive protein and procalcitonin observed during the infection course were 167.9 mg/L (mean, standard deviation [SD] 103.0) and 2.0 ng/mL (median, IQR 0.7–7.5), respectively. Both markers were detected at a median of 2 days (IQR 1–13) after symptom onset. In 15 out of 18 cases, the highest C-reactive protein level exceeded 80 mg/L, while in 15 out of 16 cases, the highest procalcitonin level exceeded 0.5 ng/mL, with 8 cases exceeding 2 ng/mL.

Six children did not require hospitalization prior to symptom onset. Ten children were admitted to the Pediatric Intensive Care Unit (PICU) at some stage during their illness, with a median stay of 5 days (IQR 2–10). The mean Pediatric Risk of Mortality (PRISM) III score was 5.1 points (SD 4.3), and the median estimated mortality according to PRISM IV was 2.5% (IQR 1.25–6.75%).

### 2.3. Microbiology and Susceptibility Profile

The predominant microorganism identified in the study was *Klebsiella pneumoniae*, with a total of 16 cases recorded, whereas *Pseudomonas aeruginosa* was isolated in just 1 instance. In another patient, carbapenem resistance mediated by KPC was detected in a blood sample when utilizing the T2 Resistance^®^ Panel from T2 Biosystems, Lexington, MA, USA, while yielding negative results in the T2 Bacteria^®^ Panel, also from T2 Biosystems, USA.

Empirical administration of CZA was initiated for eight patients prompted by rectal colonization with *K. pneumoniae*. *K. pneumoniae* was isolated from the bile (*n* = 4), tracheal aspirate (*n* = 2), liver abscess (*n* = 1), and ascitic fluid (*n* = 1) cultures in another eight patients, who were also found to be colonized with *K. pneumoniae*. Moreover, *P. aeruginosa* was cultured from a tracheal aspirate retrieved from a patient with cystic fibrosis. These clinical specimens were obtained at a median of 0 days following symptom onset, with an IQR spanning from −3 to 4 days.

The antimicrobial susceptibility profile of the *K. pneumoniae* isolates is detailed in Table 2. Among the 16 isolates, 15 were identified as OXA-48-like carbapenemase producers, with 13 of them also exhibiting ESBL production, while 1 solely displayed ESBL production. Regarding the *P. aeruginosa* isolate, no carbapenemase-related genes were detected, yet it demonstrated resistance to all the antimicrobials except for CZA, cefiderocol, ceftolozane–tazobactam, and colistin.

### 2.4. Treatment

Intravenous antibiotics were promptly initiated in all instances within the initial 24 h period following symptom onset. CZA therapy commenced at a median of 3 days (IQR 0–13) thereafter. This treatment was conducted in a hospital setting in all cases. While in 6 occurrences, CZA emerged as the initial antibiotic regimen, in the remaining 12 instances, various alternative antibiotics were judiciously prescribed. These encompassed a combination of meropenem and amikacin (*n* = 6), meropenem alone (*n* = 2), piperacillin–tazobactam (*n* = 2), a combination of meropenem and ceftazidime (*n* = 1), and a combination of meropenem and levofloxacin (*n* = 1).

The mean duration of CZA treatment was 15.7 days (SD 9.4). The dosage regimens were 50–12.5 mg/kg/dose (maximum dose 2–0.5 g) administered every 8 h for children aged ≥6 months, and 40–10 mg/kg/dose every 8 h for the single child aged <6 months. Notably, adjustments in dosage were meticulously executed in response to renal impairment, impacting the treatment of three pediatric cases.

Concomitant intravenous antibiotics targeting Gram-negative pathogens were co-administered alongside CZA for a minimum duration of 2 days in 11 pediatric patients. The administration of these adjunctive antibiotics ceased in all but three cases within less than 7 days following the conclusion of CZA treatment. Among the antibiotics frequently co-administered alongside CZA were amikacin (*n* = 6), levofloxacin (*n* = 4), colistin (*n* = 3), and meropenem (*n* = 2).

Lastly, source control surgical interventions, executed within the pivotal 7-day window post-symptom onset, were noted in two instances. Of these, only one intervention was conducted within the initial 12 h.

#### 2.4.1. Response to Treatment

CZA was generally well tolerated, and no patients discontinued treatment due to adverse events. 

Fifteen patients were discharged from the hospital after a median hospitalization duration of 77 days (IQR 32–113). Notably, the C-reactive protein levels normalized (<5 mg/L) prior to discharge in 10 patients, with normalization occurring after a median of 12 days (IQR 7–24) post-initiation of CZA therapy. The remaining three patients died before hospital discharge. Two of them (11%) died during the first month following the commencement of CZA therapy. One case involved a 2-year-old girl diagnosed with Shone complex who passed away on the eighth day of treatment, attributed to a pulmonary hypertension crisis amid resolving tracheobronchitis. Similarly, a 10-year-old boy with cystic fibrosis experienced multifactorial respiratory failure, ultimately leading to his demise on the 14th day of treatment. The presence of *P. aeruginosa* infection was directly linked to his fatal outcome. The remaining patient died more than a month after the initiation of CZA therapy from a non-infectious cause.

After a period exceeding 30 days from the initial dose, the pathogen present before treatment was once again identified at the same site in two instances, both of which had positive bile cultures. In five other cases, the pathogen was detected at a different location: specifically, in three bile cultures (involving two children who were previously colonized and one with a prior positive culture of liver abscess) and in two ascitic fluid cultures (involving one child who was previously colonized and another with a prior positive bile culture).

Following the initiation of CZA treatment, the susceptibility of *K. pneumoniae* to CZA was assessed in 26 cultures obtained subsequently, in which this microorganism was isolated again, comprising 12 rectal swabs, 10 bile samples, 2 ascitic fluid samples, and 2 blood samples, collected from 12 patients at a median interval of 103 days (IQR 42–154) post-initiation of the first dose. Notably, none of these cases exhibited the development of CZA resistance.

#### 2.4.2. Additional Courses of Ceftazidime–Avibactam Therapy and Subsequent Outcomes

New courses of therapy with ceftazidime–avibactam were administered to a subset of 10 out of the initial 18 children. A comprehensive overview of the infection type, isolates, and therapeutic regimen from these subsequent 16 occurrences is provided in Table 3.

It is noteworthy to specify, regarding patients 7, 8, 9, and 10, as they received treatment containing CZA on multiple occasions, their severity and underlying condition. In all instances, these were patients experiencing recurrent episodes of cholangitis. Two cases were infants under one year old (7 and 8) with biliary atresia treated with Kasai portoenterostomy. The other two (9 and 10) were patients in their first year post-liver transplantation complicated by bile duct strictures.

Throughout these treatment episodes, no notable adverse effects of CZA were documented. However, it is worth noting that one child (listed as number 10 in Table 3 and not included in the three previously mentioned fatalities) experienced a fatal outcome 31 days subsequent to the completion of their fifth course of CZA therapy, attributed to a non-infectious etiology. It is pertinent to mention that the remaining cases were still alive at the time of data collection. Additionally, in 10 instances, the C-reactive protein level transitioned to a negative status following a median period of 13 days (IQR 8–19) from the initiation of CZA treatment.

After completing their prescribed antibiotic course, three children, specifically identified as numbers 4, 6, and 7, underwent susceptibility testing for *K. pneumoniae* isolates to evaluate their response to CZA in subsequent new isolations. Furthermore, *K. pneumoniae* isolates were procured from patients numbered 8 and 10 after 21 and 118 days of receiving therapy, respectively, and subsequently subjected to susceptibility testing for CZA. It is important to highlight that, as previously stated, none of these cases exhibited the emergence of resistance to the antibiotic.

## 3. Discussion

This study presents the most extensive collection of CZA-treated episodes in pediatric patients documented thus far. The study population consisted of a cohort of 18 children with a spectrum of complex chronic conditions, most of whom presented with intra-abdominal infections attributed to or suspected to be caused by OXA-48-producing *K. pneumoniae*. The administration of CZA was met with favorable tolerance, and one mortality incident within the initial month of therapy was directly linked to the infection. Notably, a subset of 10 patients required multiple treatment courses owing to recurrent infections; remarkably, no instances of CZA resistance emerged.

Significant strides in neonatal, medical, and surgical care have markedly elevated the survival rates of individuals grappling with severe health challenges, thereby resulting in a pronounced rise in the prevalence of children afflicted with complex chronic conditions and reliant on medical technology [12]. This surge in complex medical needs has subsequently contributed to a concerning escalation in the incidence of healthcare-associated infections, heightened antibiotic usage, and an uptick in infections caused by multidrug-resistant organisms [13]. As anticipated, every participant in our study presented with at least one pediatric complex chronic condition. Notably, eight of these individuals had undergone liver transplantation, a procedure well-documented as a predisposing factor for infection by CR *Enterobacterales* [14].

Despite the endorsement of pediatric usage by both the FDA and EMA, the available data on the application of CZA in pediatric patients with cIAI remain notably sparse. To date, only two studies have been published on this topic. The initial study was a phase II trial involving pediatric patients diagnosed with cIAI unrelated to multidrug-resistant organisms, primarily focusing on individuals aged over 6 years with appendicitis [15]. In our series, which comprised 18 patients, the majority of whom were younger and presented with more complex medical conditions, a total of 34 courses of therapy were administered, with 29 of these courses being directed toward gastrointestinal infections associated with MR *K. pneumoniae*. This scenario likely offers a more accurate reflection of the clinical context wherein the utilization of CZA is necessary. Recently, Wang et al. published a notable series comprising six cases of pediatric patients who underwent liver transplantation and presented with cIAI, all of which were effectively managed using CZA [16]. Furthermore, data on the off-label utilization of CZA in pediatric patients for conditions such as respiratory tract and bloodstream infections, accounting for five treatment courses, as well as its application in critically ill pediatric cases (comprising 10 admissions to the PICU), remain limited [17,18,19,20,21,22].

One death was directly attributed to the infection necessitating the initiation of CZA, and multiple recurrences were noted within our study cohort. It is challenging to draw direct comparisons with the existing literature in children, as such adverse events have been scarcely documented. This discrepancy may be attributed to notable differences in the baseline characteristics [15,22,23] and infection types [17,21,22]. While the patient demographics and types of infections in our study could be somewhat comparable to the previously mentioned study by Wang et al., unlike our approach, where we investigated the recurrence of infections caused by CR *Enterobacterales* beyond the hospitalization period, Wang et al.’s study solely concentrated on assessing recurrence during the hospital stay [16]. Therefore, the drawing of conclusive comparisons between the two studies may be limited.

The extensive dispersion of OXA-48 producers throughout Western Europe underscores why the vast majority of carbapenemase producers identified in our study (15 out of 16) were OXA-48-like producers [24]. A study from 2016, spanning five tertiary hospitals in Spain, which included our own, reported a prevalence rate of 1.6% for carbapenemase-producing *Enterobacterales* in urine samples. Notably, *K. pneumoniae* (87.8%) emerged as the most commonly encountered pathogen, with OXA-48 (86.8%) standing out as the predominant carbapenemase variant [25].

CZA has consistently demonstrated exceptional in vitro efficacy, ranging from 99.8% to 100%, against carbapenemase-positive, metallo-beta-lactamase (MBL)-negative *Enterobacterales* in various research studies [25,26,27]. Despite this, the emergence of resistance during CZA therapy remains a significant concern. This resistance is most commonly associated with KPC-2- and KPC-3-producing isolates, even after a relatively brief treatment period [28,29]. The most informative insights originate from extensive case series involving adult patients, where the susceptibility of CZA was meticulously examined in individuals experiencing recurrent infections attributed to carbapenemase-producing *Enterobacterales*. Notably, these investigations were predominantly undertaken within the United States, where KPC stands out as the prevailing carbapenemase in such clinical scenarios.

The two most expansive series examining the emergence of resistance among OXA-48-producing *Enterobacterales* treated with CZA, both conducted in Spain, solely assessed the MIC values in three and six instances of recurrent infections, wherein the isolates remained susceptible [30,31]. The complex and chronic nature of our study population not only elucidates the persistent presence of risk factors for CR infection or colonization but also underscores the challenges in attaining source control objectives. Consequently, a substantial number of recurrent infections ensued, necessitating multiple courses of therapy, affording us the opportunity to investigate the potential emergence of resistance among OXA-48-producing isolates within a vulnerable demographic. However, notably, resistance did not manifest in any of the 12 patients who exhibited positive *K. pneumoniae* cultures following treatment with CZA, highlighting its infrequent occurrence and representing a significant finding with profound implications for both pediatric and adult populations.

The findings of our study offer valuable insights into the appropriate use of CZA in children, contributing to the collective understanding of the optimal treatment strategies. However, it is imperative to acknowledge and scrutinize several potential limitations inherent to the study design. Firstly, owing to its retrospective nature, the accuracy and comprehensiveness of the clinical data are contingent upon the thorough review of medical records, which may introduce inherent biases or inaccuracies. Secondly, if an expert in antibiotic use had been involved in all the cases, treatment with CZA might have been optimized for some patients, potentially resulting in fewer, shorter, and/or more effective courses of therapy. Thirdly, we lack confirmatory evidence that the eight patients who were treated based on rectal colonization had an MR infection. This is not surprising, as empirical treatment with ceftazidime–avibactam in patients colonized by multidrug-resistant Gram-negative bacteria with a severe infection is a common practice in clinical settings, as demonstrated by the study conducted by Ftergioti et al., who investigated the use of this antibiotic in a neonatal intensive care unit [22]. However, it is noteworthy that a high proportion of colonized children (13%) subsequently developed infections caused by CR *Enterobacterales* in two separate studies [32,33]. Furthermore, colonization with carbapenemase-producing *Enterobacterales*, either before or after liver transplantation, has been shown to be a strong predictor of carbapenemase-producing *Enterobacterales* infection in adults [34]. Additionally, the relatively small sample size is a notable limitation, which may reduce the statistical power of the study and affect the reliability of the results. Finally, adverse effects were not systematically recorded and, if present, their cause could have been difficult to interpret.

Future research endeavors should delve into elucidating the most effective empirical and targeted treatment modalities for CR *Enterobacterales* infections in the pediatric population. While CZA may emerge as a promising first-line therapy option for OXA-48 producers, the emergence of resistance among KPC producers during treatment underscores the necessity of exploring novel therapeutic strategies. This warrants investigation into the efficacy and safety profiles of emerging antibiotics such as cefiderocol, meropenem–vaborbactam, or imipenem–relebactam in pediatric populations. Additionally, there is a pressing need for studies focusing on dose optimization guided by pharmacokinetic/pharmacodynamic (PK/PD) principles and the exploration of combination therapy approaches. Clinical trials are imperative to evaluate the safety and efficacy of CZA in both term and preterm neonates, as well as its potential in off-label indications currently lacking comprehensive evidence.

## 4. Materials and Methods

### 4.1. Study Design and Approvals

We conducted a descriptive case series utilizing data extracted from the medical records of pediatric patients spanning from infancy to adolescence, encompassing ages ranging from 0 to 16 years, who were under the care of La Paz University Hospital, located in Madrid, Spain, throughout the period extending from February 2019 to January 2022. La Paz University Hospital boasts a collective capacity of 280 beds designated for pediatric patients, including 23 specifically designated for neonatal care and an additional 16 designated for pediatric intensive care units. Renowned as a national referral teaching hospital, it hosts all the pediatric solid organ transplant programs. Furthermore, our institution pioneered the establishment of a dedicated unit for managing pediatric complex chronic conditions in 2008, marking a significant milestone as the first of its kind in Spain. It is important to note that in the year 2019, our hospital observed a shift in the trend regarding the most frequently encountered carbapenemase type in children colonized by MR Gram-negative bacteria. Prior to this year, VIM was predominant, whereas from 2019 onwards, OXA-48 became predominant [35].

This study received approval from our hospital’s institutional review board under protocol number PI-5168.

### 4.2. Study Population

The eligibility criteria encompassed patients aged below 16 years upon admission who underwent treatment with CZA for a minimum duration of 2 days, equivalent to 6 doses if administered every 8 h, or, in cases of renal impairment, 4 doses if administered every 12 h, and 2 doses if administered every 24 h.

Prior to the initial implementation of CZA in February 2019, our hospital had initiated an antimicrobial stewardship program, which was operational in select departments. Within this framework, a thorough daily assessment of antibiotic therapies for pediatric patients admitted to the PICU was conducted by an expert in antibiotic utilization. Feedback sessions were conducted on a face-to-face basis with intensivists, offering personalized insights. Prescribing physicians were granted the autonomy to utilize CZA at their discretion, contingent upon prior confirmation of susceptibility. Given that the study period coincides with the COVID-19 pandemic, we find it important to clarify that CZA was not used in our hospital at any time for COVID-19 infections. Throughout the pandemic, its use was strictly limited to infections caused by MR Gram-negative bacteria.

### 4.3. Data Sources and Measures

Patients who had undergone treatment with CZA were identified by accessing the Hospital Pharmacy Department database. The selection process focused on individuals aged below 16 years who met the predefined inclusion criteria. A comprehensive set of data, encompassing demographics, risk factors, clinical manifestations and severity, laboratory findings, microbial isolates, details of CZA treatment (including dosage and duration), concomitant administration of other antimicrobial agents, adverse events, as well as microbiological and clinical responses and outcomes, was meticulously abstracted from the medical records. To ensure thoroughness, the causes of mortality were meticulously reviewed by two investigators.

Our analysis involved an assessment of the underlying medical conditions and the presence of risk factors associated with CR infection or colonization at the onset of symptomatic presentation, leading to the initiation of the first course of CZA therapy. Utilizing the pediatric complex chronic conditions classification system version 2 developed by Feudtner et al. [36], we categorized the underlying conditions observed in the pediatric cohort. Moreover, adherence to the CDC/National Healthcare Safety Network (NHSN) surveillance definition of healthcare-associated infections, alongside the criteria delineated for specific infection types in the acute care setting, facilitated the classification of the clinical entities necessitating treatment with CZA [37]. Given the high complexity of our patient population, the risk they faced in case of delayed antibiotic treatment, as well as the difficulty in obtaining optimal microbiological samples, especially among those with liver transplants and biliary atresia, it was expected that some may not meet the criteria for any specific infection; for these cases, where the criteria for a specific infection were not entirely met, we interpreted the most likely type as probable rather than proven. The definitions of sepsis and septic shock were in accordance with the parameters outlined by the International Consensus Conference on Pediatric Sepsis [38].

The severity of illness was evaluated by employing the PRISM III and IV scoring systems. These assessments encompassed the measurement of physiologic variables during the initial 4 h following admission to the PICU, coupled with the monitoring of laboratory parameters from 2 h preceding PICU admission up to the first 4 h [39,40]. The adverse events linked to CZA were documented based on the interpretation provided by the attending physicians responsible for patient care.

Rectal colonization was defined by the detection of a positive result from a rectal swab culture conducted within the 15-day period prior to the administration of the initial dose of CZA. The determination of antimicrobial susceptibilities was carried out using an automated broth microdilution assay system (Microscan Walkaway^®^, Beckman Coulter, Brea, CA, USA), with interpretations performed in accordance with the guidelines outlined by the European Committee on Antimicrobial Susceptibility Testing (EUCAST) [41]. ESBL production was identified through the same broth microdilution assay. In cases where the minimum inhibitory concentration (MIC) of ertapenem exceeded 0.5 mg/L, confirmation of carbapenemase production (OXA-48, VIM, KPC, NDM) was conducted via molecular methodology utilizing OXVIKPND^®^ real-time PCR kits (Progenie Molecular, Valencia, Spain).

### 4.4. Statistical Analysis

Quantitative variables that followed a normal distribution were presented using the mean and SD, while non-normally distributed variables were represented by the median and IQR. The normality of the quantitative variables was assessed using the Shapiro–Wilk test. Statistical analysis was conducted using the SPSS program version 20.0.

## 5. Conclusions

In conclusion, based on our comprehensive case series involving pediatric patients with complex chronic conditions and MR infections, mostly intra-abdominal infections attributed to or suspected to be caused by OXA-48-producing *K. pneumoniae*, the utilization of CZA demonstrated both efficacy and safety. Despite encountering frequent episodes of recurrent infections necessitating multiple courses of therapy, the notable absence of resistance development underscores the potential of CZA as a frontline therapeutic option for combating infections caused by OXA-48 producers.

## Figures and Tables

**Table 1:** Demographics, risk factors, and clinical illness of 18 children receiving a first course of therapy with ceftazidime–avibactam.

	N	%
Patients having at least 1 PCCC	18	100
Total number of PCCC, median (IQR)	1.5 (1–3)	
Primary underlying condition		
Liver transplant status	8	44
Biliary atresia	4	22
Mechanical ventilation through tracheostomy in congenital heart disease	3	17
Intestinal epithelial dysplasia	1	6
Hematopoietic stem cell transplant	1	6
Cystic fibrosis	1	6
Risk factors for carbapenem-resistant infection or colonization		
Presence of central venous catheter	14	78
Use of antibiotics in the previous 90 days		
Carbapenems	13	72
3rd- and 4th-generation cephalosporins	7	39
Aminoglycosides	7	39
Presence of gastrointestinal medical devices	12	67
Nasogastric tube	10	56
Surgical drainage	5	28
Major surgery in the previous 90 days	10	56
Length of hospital stay >21 days	6	33
Mechanical ventilation	3	17
Type of infection		
Probable intraabdominal infection	8	44
Proven intraabdominal infection	5	28
Proven tracheobronchitis	2	11
Probable pneumonia	1	6
Gastroenteritis	1	6
Laboratory-confirmed bloodstream infection	1	6
Fever	11	61
Duration of fever (days), median (IQR)	2 (1–3)	
Sepsis	12	67
Septic shock	7	39

Abbreviations: IQR = interquartile range; PCCC = pediatric complex chronic condition.

**Table 2:** Antimicrobial susceptibility of 16 isolates of *Klebsiella pneumoniae*.

	Antimicrobial Susceptibility *		
Antibiotic	S	I	R
Ampicillin	0	-	16
Piperacillin	0	-	16
Piperacillin–tazobactam	1	-	15
Cefepime	0	0	16
Cefotaxime	0	0	16
Ceftazidime	0	0	16
Ceftazidime–avibactam	16	-	0
Ertapenem	1	-	15
Imipenem	4	2	10
Meropenem	7	1	8
Aztreonam	0	0	16
Ciprofloxacin	1	0	15
Levofloxacin	5	5	6
Amikacin	6	-	10
Gentamicin	0	-	16
Tobramycin	0	-	16
Tigecycline	10	2	4
Colistin	14	-	2
Fosfomycin iv	2	-	14
Trimethoprim–sulfamethoxazole	1	0	15

Abbreviations: S = susceptible, standard dosing regimen; I = susceptible, increased exposure; R = resistant. * Source of antimicrobial susceptibility interpretation: The European Committee on Antimicrobial Susceptibility Testing. Breakpoint tables for interpretation of MICs and zone diameters. Version 12.0, 2022. Available at: http://www.eucast.org (accessed on 1 June 2022).

**Table 3:** Overview of infection types, isolates and therapeutic regimens in 10 children undergoing multiple courses of ceftazidime–avibactam therapy.

Patient No.	Number of Courses of Therapy	Infection Type	Pathogen	OXA-48-like Carbapenemase Producer	ESBL Producer	Specimen	Duration of 1st Course of CZA (days)	Days of Therapy with CZA after 1st Course
1	2	Probable IAB	*K. pneumoniae*	Yes	Yes	Rectal swab	19	9
2	2	Probable IAB	*K. pneumoniae*	Yes	Yes	Bile	21	13
3	2	GIT	*K. pneumoniae*	Yes	Yes	Rectal swab	4	15
4	2	LCBI	*K. pneumoniae*	Yes	Yes	Blood	2	17
5	2	Probable IAB	*K. pneumoniae*	Yes	Yes	Bile	16	18
6	2	IAB	*K. pneumoniae*	Yes	Yes	Ascitic fluid	33	21
7	3	Probable IAB	*K. pneumoniae*	Yes	Yes	Rectal swab	11	21
8	3	Probable IAB	*K. pneumoniae*	Yes	No	Rectal swab	7	29
9	3	IAB	*K. pneumoniae*	Yes	Yes	Bile	22	53
10	5	IAB	*K. pneumoniae*	Yes	Yes	Bile and Ascitic fluid	27	146

Abbreviations: CZA = ceftazidime–avibactam; ESBL = extended-spectrum beta-lactamase; GIT = gastrointestinal tract excluding gastroenteritis and appendicitis; IAB = intraabdominal infection(s), not specified elsewhere; LCBI = laboratory-confirmed bloodstream infection.

## Data Availability

Due to privacy and ethical restrictions, the data used in this study are not publicly available. However, they are available upon reasonable request to the corresponding author, subject to privacy and data protection policies.
